# Isolation and Characterization of a Dominant Dwarf Gene, *D-h*, in Rice

**DOI:** 10.1371/journal.pone.0086210

**Published:** 2014-02-03

**Authors:** Rihua Piao, Sang-Ho Chu, Wenzhu Jiang, Yoye Yu, Yongmei Jin, Mi-Ok Woo, Joohyun Lee, Sunghan Kim, Hee-Jong Koh

**Affiliations:** 1 Department of Plant Science, Research Institute of Agriculture and Life Sciences and Plant Genomics and Breeding Institute, College of Agriculture and Life Sciences, Seoul National University, Seoul, Korea; 2 College of Plant Science, Jilin University, Changchun, China; 3 Agro-Biothechnology Research Institute, Jilin Academy of Agricultural Sciences, Changchun, China; 4 Department of Applied Bio Science, Konkuk University, Seoul, Korea; Universidad Miguel Hernández de Elche, Spain

## Abstract

Plant height is an important agronomic trait that affects grain yield. Previously, we reported a novel semi-dominant dwarfmutant, HD1, derived from chemical mutagenesis using N-methyl-N-nitrosourea (MNU) on a *japonica* rice cultivar, Hwacheong. In this study, we cloned the gene responsible for the dwarf mutant using a map-based approach. Fine mapping revealed that the mutant gene was located on the short arm of chromosome 1 in a 48 kb region. Sequencing of the candidate genes and rapid amplification of cDNA ends-polymerase chain reaction (RACE-PCR) analysis identified the gene, *d-h*, which encodes a protein of unknown function but whose sequence is conserved in other cereal crops. Real-time (RT)-PCR analysis and promoter activity assays showed that the *d-h* gene was primarily expressed in the nodes and the panicle. In the HD1 plant, the *d-h* gene was found to carry a 63-bp deletion in the ORF region that was subsequently confirmed by transgenic experiments to be directly responsible for the gain-of-function phenotype observed in the mutant. Since the mutant plants exhibit a defect in GA response, but not in the GA synthetic pathway, it appears that the *d-h* gene may be involved in a GA signaling pathway.

## Introduction

Dwarf and semi-dwarf characteristics are important agronomic traits in rice breeding forlodging resistance and higher yields. The introduction of dwarfing genes through breeding programs was instrumental in the ‘green revolution’ in cereals [1]. In rice, the semi-dwarf varieties thathave been developed since the 1960s and carry the recessive semi-dwarf gene 1(*sd1*) have delivered significant yield improvements [2].

Dwarfism in plants can be caused by defects in various hormones, but only the defects in brassinosteroids (BR) and gibberellins (GA) have been studied extensively, from which many dwarf genes were identified as being related to the biosynthesis or signaling of these phytohormones [3], [4]. GAs are a group of diterpenoid compounds that act asregulators of vegetative and reproductive development, including stem elongation, leaf differentiation, photomorphogenesis, pollen development, and flowering. The characteristic phenotype of the GA-related mutants shows deep green leaves but no other abnormal morphologies except dwarfism [4]–[7]. Brassinosteroids (BRs), a class of plant steroid hormones, mediate many important processes in plant growth and development, including the stimulation of cell division, cell elongation, vascular differentiation, and stress response. BR-related mutants usually exhibit pleiotropic phenotypes, including compact stature, deep green and erect leaves, altered organ size, internode elongation in the dark, and delayed flowering [3], [8]–[10].

More than 60 dwarf mutants have been reported in rice (http://www.gramene.org/rice_mutant/). However, a majority of these are caused by recessive alleles with impractical pleiotropic phenotypesexerting unfavorable effects on many agronomic characteristics. Therefore, only *sd1* and its allelic mutants have been widely used in rice breeding.The extensive use of limited dwarfing sources may cause a bottleneck effect in the genetic background for new rice varieties, and identifying and developing new useful dwarfs is therefore an important subject for practical rice breeding [2].

The incorporation of the dwarfing gene into a rice breeding program can be facilitated by the use of a dominant allele that can avoid the masking of the trait in the F_1_ hybrid [11]. Some dominant or semi-dominant rice mutants have been reported previously, including *D53*,*Ssi1*, *Sdd(t)*, *Dx*, *TID1*, *LB4D*, *Slr-d*, and *D-h*, which were developed in our laboratory using chemical mutagenesis [11]–[18]. *D-h* was characterized by shortcompact panicles and smallround grains, both of which are likely controlled by a single dominant allele [12]. In this paper, we report the map-based isolation of the *D-h* gene and the identification of a 63-bp deletion in the corresponding locus of the *d-h*. Identification of this gene and its mutant might lead to the isolation of a new functional protein responsible for smallround grains and semi-dwarf phenotypes.

## Materials and Methods

### Plant materials

A dominant dwarf mutant line was induced by the chemical mutagenesis of a*japonica* rice cultivar,wild typeHwacheong (WT), using N-methyl-N-nitrosourea, and were propagated for several generations to obtain stable lines in the greenhouse and/or experimental field. The seeds of the dwarf Hwacheongmutant (designated HD1) used in this study were taken from the M_13_ generation. HD1(MT)was crossed with Milyang 23 (*indica*, PI 464609), Tetep(*indica*, PI 431324), and Hwacheong (*japonica*). Two of the HD1×Milyang 23 and HD1×Tetep F_2_ populations and three of the segregating F_3_ lines from the cross between HD1 and Milyang 23 were used for fine mapping and gene cloning. In order to confirm the heterozygosity of the selected F_2_ plants, the F_3_ progeny were observed for phenotypic segregation. An F_2_ population from the cross between the HD1 and wild-typeHwacheongbyeo was used for the insertion-deletion(InDel) marker analysis. All of the plant materials for gene cloning were grown at the experimental farm of the Seoul National University, Suwon, Korea, in 2007, 2008, and 2009.

### Assay of α-amylaseactivity

Ten embryoless half-seeds were hulled, surface sterilized for 30 min with a 3% NaClO solution, and washed three times with sterile distilled water. The half-seeds were then placed on 2% agar plates containing 10 mM sodium acetate and 2 mM CaCl_2_ at a pH of 5.3.Gibberellic acid (GA)supplementation was achieved by adding 0–10^−4^ M GA_3_. The activity of α-amylase was measured using a method modified slightly from that of Okamoto and Akazawa [19]. Specifically, plates were incubated for 4 days at 30°C in darkness and placed in a box saturated with iodine vapor. The half-seeds that synthesized and secreted α-amylase were characterized by clear zones around them on the plate, resulting from the digestion of the starch by their secreted α-amylases.

### GA induction in shoot elongation

In order to investigate the role of GA in second leaf sheath elongation, 10 rice seeds were sterilized with 3% NaClO for 30 min, washed five times in sterile distilled water, and then incubated at 30°C for 2 days. The seeds were then placed on 1% agar plates containing various concentrations of GA and were incubated at 30°C under continuous light. After 6 days of incubation, the length of the second leaf sheath was measured.

### Paraffin embedded sections of tissues

For paraffin sectioning, the tissues were prepared as described previously [20]with slight modifications. The leaf, stem, root, and panicle were harvested at the ripening stage. The samples were sectioned at a thickness of 10 µm using a rotary microtome (MICROM Lab, Walldorf, Germany). The prepared samples were observed under a light microscope at 100× magnification.

### PCR-based markers for physical mapping and InDel maker analysis

For fine mapping, 12 sequence-tagged site (STS) markers were developed based on the differences in the DNA sequences between the *indica* and *japonica* rice subspecies (http://www.ncbi.nlm.nih.gov/ for *indica* and http://www.rgp.dna.affrc.go.jp/ for *japonica*). An InDel marker was designed based on the sequence alignment of the candidate gene between the original Hwacheong and HD1. The primer sequences of the STS markers and theInDelmarker used in this study are listed in Table S1. Linkage analysis was conducted using MAPMAKER version 3.0 [21]and QTL Mapper2.0 [22].

### RACE-PCR

Genomic sequences were searched for the predicted ORFs using FGENESH [23]and GENESCAN [24]. Subsequently, putative ORF sequences were used to search GenBankusing BLAST. 5′ and 3′ rapid amplification of cDNA ends (RACE) by PCR was performedusing the SMART RACE cDNA amplification kit (BD Biosciences Clontech) and RNA extracted from the WT and HD1inflorescences as template. RACE was performed according to the manufacturer's instructions.First-strand cDNAsynthesis was primed with SMART universal primers. The gene-specific primers 5-GSP1 (5′- ATGGCGCGGTCCTCGGCCGC-3′) and 5-GSP2 (5′-CTTGAACGACGTGGTGTCC-3′) were used for 5′ RACE and the gene-specific primers 3-GSP1 (5′-GGACACCACGTCGTTCAAG-3′) and 3-GSP2 (5′-GCGGCCGAGGACCGCGCCAT-3′) were used for 3′ RACE. The final RACE products were subcloned and sequenced.

### RNA isolation and quantitative real-time RT-PCR

Total RNA was extracted from different tissues of the WT and HD1 at the heading stage using Trizol reagent (Invitrogen, USA). Total RNA was treated with DNase (RNase-free DNase set, Takara, Japan) and reverse transcription was performed with a ReverTraAce ® qPCR RT Kit (Toyobo, Japan). The primers used were as follows: 5′-GCGGCCGAGGACCGCGCCAT-3′ and 5′-CTTTAAGCTCGGCGATCAATTAATC-3′ for *d-h* and 5′-TGTCATGGTTGGAATGGGCCA-3′ and 5′-AGGCAGTCAGTCAGATCACGA-3′ for actin. Real-time PCR was performed with a C1000 thermal cycler, (Bio-Rad, USA).

### Subcellular localization of the *D-h* protein

The amplified predicted coding regions of the *D-h* gene from both WT and HD1 were cloned into the PCR/GW/TOPO vector (Invitrogen) and then inserted into the pMDC83 gateway binary vector [21]. The expression constructs were bombarded into onion epidermal cells using a PDS-1000/He particle gun (Bio-Rad). Twenty hours after transformation, GFP (green fluorescence protein) fluorescence was examined with image restoration microscopy (Delta Vision RT, Applied Precision).

### Vector constructs and rice transformation

In order to generate overexpression vectors, PCR-amplified WT and HD1 full-length cDNAs were digested with KpnI and XbaI and then inserted into the pCAMBIA 1300-modified vector containing a 35S promoter and anos terminator. The resulting WT cDNA overexpression construct was denoted 35s::*d-h*-W and the mutant cDNA construct was denoted 35S::*D-h*-M. In order to generate the RNAi::*d-h*-W construct for *D-h* gene suppression, a 336 bp fragment of *d-h*cDNA spanning nucleotides 217 to 553 was first cloned into pDONR201 (Invitrogen)and then cloned in sense and antisense directions into the binary transformation vector pH7GWIWG(II)using the Gateway BP and LR clonase enzyme mixes(Invitrogen). pH7GWIWG(II) and derivatives contain the hygromycin resistance (Hyg) gene. For the promoter-GUS assay, the genomic sequence containing the putative promoter region of *D-h* (−2234 to −1 bp from the translation initiation codon) was amplified by PCR from the genomic DNA. The *D-h* promoter fragment was cloned into the binary vector pHGWFS7. Transgenic plants carrying the above constructs were generated using wild-type Dongjin (a *japonica* cultivar) seeds and HD1 seeds via agrobacterium-mediated co-culture methods [25].

## Results

### Characterization of the dominant dwarf mutant

**Figure 1:**
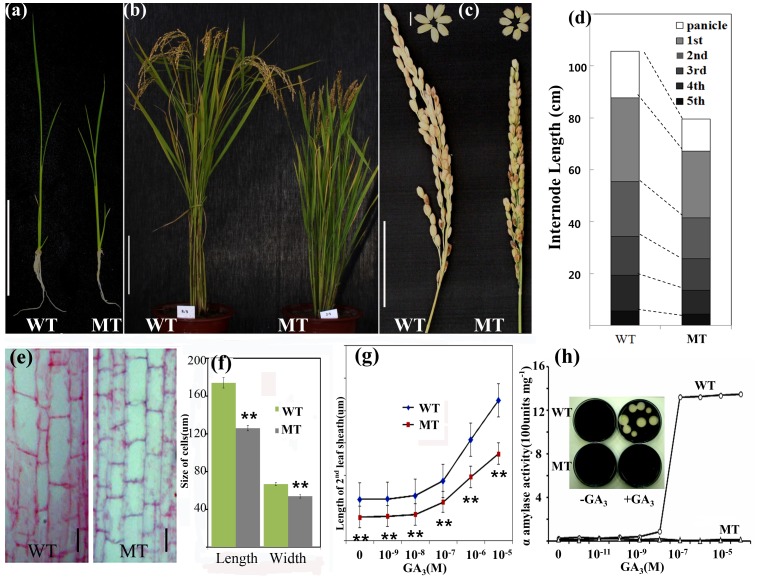
Characterization of the HD1. (a) Seedling phenotype of the HD1 and WT. Bar = 5 cm. (b) The HD1 and WT at 3 weeks after the heading stage. Bar = 20 cm. (c) Mature panicle and seeds of the HD1 and WT. Bottom bar = 5 cm (panicle), top bar = 5 mm (seeds). (d) Internode lengths of the HD1 and WT. The average values were calculated from measurements of at least 10 plants. (e) Parenchyma cells in the first internode in the HD1 and WT. Bars = 50 µm. (f) Quantitative measurements of the cell length and cell width of the HD1 and WT (n = 20). Data are mean±SD. Asterisks indicate a significant difference at P≤0.01 compared with the WT by Student's t test. (g) Induction of α-amylase activity by gibberellic acid (GA_3_) in the HD1 and WT. (h) Elongation of the second leaf sheath in the HD1 and the WT in response to GA_3_. Data are mean±SD (n = 10). Asterisks indicate a significant difference at P≤0.01 compared with the WT by Student's t test.

The morphologies of WT and HD1 plants are shown in Figure 1 (a–b). The mutant was shorter than the WT at both the seedling stage and the grain-filling stage. In addition, the mutant spikelets and grains were noticeably shorter than those of the WT in all the mutant population we examined (Fig. 1c). Atthe heading stage, HD1plants were 74–78% of the height of the WT plants. The length of the internodes between the two plant typeswas compared, and all of the internodes of the HD1 were shorter than those of the WT. Rice dwarf mutants were previously categorized by Takeda [26], according to the elongation pattern of the internodes, into six groups: N-, dn-, dm-, d6-, nl-, and sh-. Of these, the dn-type was defined as having a reduction in the length of all of the internodes; thus, according to this scheme, the HD1is adn-type dwarf mutant (Fig. 1d).

Microscopic observation of the stem, leaf, and root cross sections showed that the overall development of the cells and tissues was not significantly different between the WT and HD1 (data not shown); however, the longitudinal sections of the uppermost internodes in the HD1 showed smaller parenchyma cells that were reduced in length and width compared with those of the WT plants (Fig. 1e and f).

In order to determine whether the dwarf phenotype of the mutant plants was caused by GA_3_ deficiency or insensitivity, we examined the effects of exogenous GA on second leaf sheath growth and induction of endosperm α-amylase activity. Varying concentrations of GA_3_, ranging from 10^−9^M to 10^−5^M, were applied to both the WT and mutant plants. A marked increase in the length of the second leaf sheath was observed in the mutant plants upon treatment with GA_3_ at concentrations between 10^−7^M and 10^−5^M. However, the rate of increase was not significantly different from that of the WT leaf sheath subjected to the same conditions (Fig. 1g), indicating that dwarfism of the HD1 is not likely to be associated with a deficiency in the endogenous GA levels. In order to further characterize the possible involvement of the *D-h* gene in the GA response, α-amylase activity was assayed in WT and mutant embryoless half-seeds on GA-containing agar plates. HD1s do not exhibit any α-amylase activity in response to the GA treatment, in contrast to the WT embryoless half-seeds (Fig. 1h), indicating that the HD1 might be defective in the GA signaling pathway.

### Molecular cloning of the *D-h* gene

**Figure 2:**
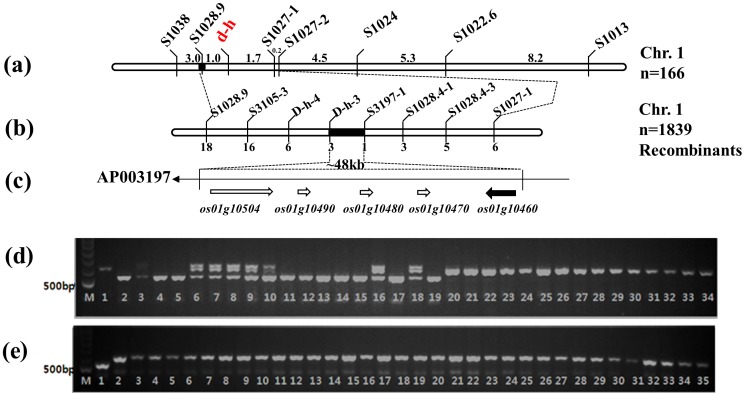
Positional cloning of the *D-h* gene. (a) Genetic mapping of the *D-h* locus with STS markers. (b) Fine mapping of the *D-h* locus with additional STS markers. (c) Candidate gene in the 48 kb genomic DNA region identified by fine mapping. (d) Co-segregation analysis in F_2_ plants of the HD1×Hwacheongbyeo cross using STS marker S10460. A 583 bp PCR product was observed in the tall homozygotes, whereas a shorter 520 bp PCR product was observed in the dwarf homozygotes. In dwarf heterozygotes, both fragments were observed. Lane 1, Hwacheong; Lane 2, HD1; Lane 3, HD1×HwacheongF_1_; Lanes 4–19, dwarf phenotype, Lanes 20 to 34, tall phenotype. (e) Genotype of the STS marker S10460 among the HD1 and 33 rice cultivars having normal clum length. Lane 1, HD1; Lanes 2–34, rice cultivars.

The HD1was obtained by chemical mutagenesis and was first reported in our previous publication [12], which described its dominant phenotype of short panicle and smallround grains in addition to dwarfism.This causal gene differs from the loci previously reported for the dominant dwarf trait in rice, thus it was designated as *D-h*. In the present study, we mapped the *D-h* locus using a population derived from a cross between HD1 and Milyang23 (*indica*). The*D-h* gene was initially traced to the short arm of chromosome 1 by bulked segregant analysis (BSA), which identified the S1022.6 and S1013 STS markers that were linked to the locus. Subsequently, a total of 166 segregated dwarf phenotype individuals from 801 F_2_ plants were used for primary gene mapping. Linkage analysis showed that the *D-h* locus was located between the STS markers S1027.3 and S1028.9 on chromosome 1 (Fig. 2a). In order to further map the *D-h* locus, a large F_3_ population derived from the original F_2_ population and another F_2_ population derived from a cross between the HD1and Tetep (*indica*) were used. We designed six STS markers after examining the genomic sequences in the target region of Nipponbare (*japonica*) and 9311 (*indica*) using rice genomic databases (http://www.ncbi.nlm.nih.gov/ for *indica* and http://www.rgp.dna.affrc.go.jp/ for *japonica*) to identify additional markers closely linked to the *D-h* locus. Subsequent linkage analysis with the eight markers and 1,839 F_3_ and F_2_ individuals revealed that the *D-h* gene was flanked by STS markers S3197-1 and D-h-3. The physical distance between these two markers was 48 kb in Nipponbare BAC clone AP003197 (Table S1, Fig. 2b and c). Five predicted geneswere located in this region (Gramene, www.gramene.org; Fig. 2c).

In order to identify the best candidate for *D-h*, we sequenced all five of the candidate genes in the HD1 and in WT and compared them with the corresponding regions in the publicly available genome sequence of cv. Nipponbare. No sequence difference between WT and HD1was noted with the exception of a region in LOC_Os1g10460(B1015E06.28). The locus was predicted to contain a 3,113 bpgenecomprising four exons and three introns. A 63-bp deletion between nucleotides 203–265 in the putative first exon was found in the HD1, as well as a single base substitution elsewhere within the same exon.

In order to determine whether the 63-bp deletion was present as a natural variant in other cultivars, we performed InDel marker analysis of 30 typical *Oryza sativa* (21 *japonica* and nine*indica*) and 4 *O. rufipogon* accessions. None of these exhibited the 63-bp deletion. In addition, we found that the genotype of the InDel marker co-segregated with the dwarf phenotype in the F_2_ population (Fig. 2d), supporting that the 63-bp deletion identified in the LOC_Os01g10460 gene was responsible for the dwarf phenotype of the HD1.

### Determination of the D-h gene transcript orientation by RACE-PCR

**Figure 3:**
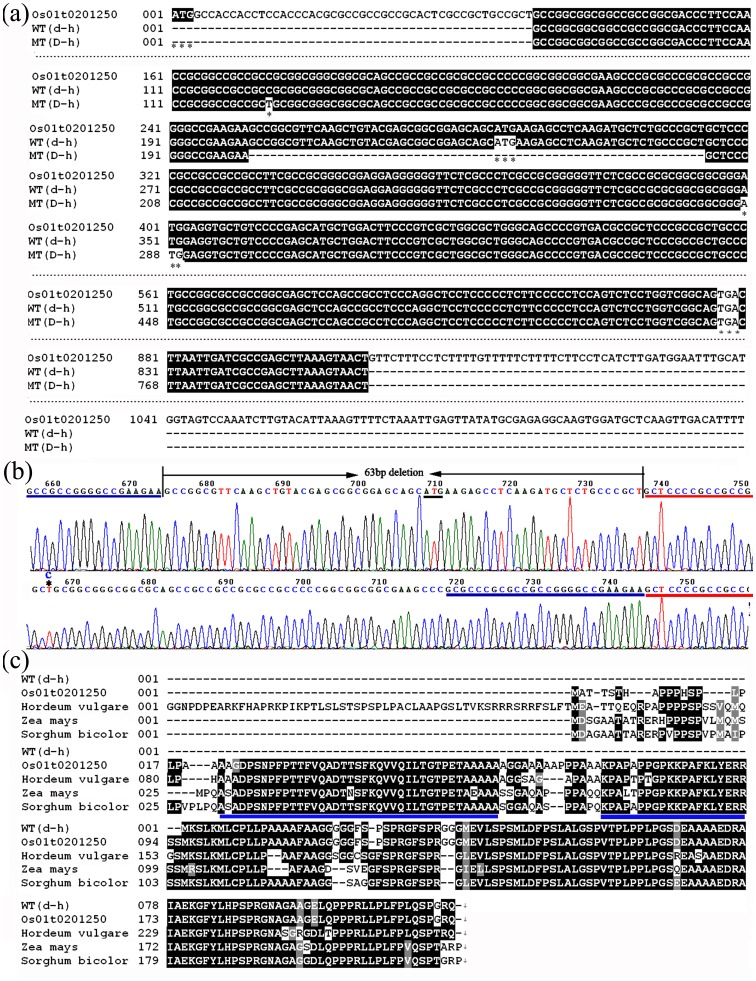
Comparison of the cDNA and predicted amino acid sequence alignments of *d-h*(WT)and*D-h*(MT). (a) Comparison of the cDNA sequences *d-h*(WT)and*D-h*(MT). Asterisks indicate single nucleotide substitution, *d-h* start codon, *D-h* start codon, and stop codon. (b) Sequence ekectrophoregrams of the RT-PCR products of *d-h*(WT)and*D-h*(MT). (c) Alignment of the predicted *d-h*protein with hypothetical proteins from *Zea mays* (NP_001147534), *Sorghum bicolor* (XP_002454989), and *Hordeumvulgare* gene (BAJ91554, AK360345).

A number of expressed sequences were identified in the region overlapping the 63-bp deletion sequences. In order to determine the precise architecture of the *D-h* gene, first FGENESH [23] and then GENESCAN [24]were used to identify potential genes by computer-aided gene prediction. Two potential genes of opposite orientations,ORF1 and ORF2, were predicted that include the mutation site. ORF1 corresponds to a transcript (Os01t0201250) annotated in the RAP-DB (http://rapdb.dna.affrc.go.jp/) andORF2 corresponds to the gene for LOC_Os01g10460 annotated in the Gramene database (www.gramene.org); these are predicted to be transcribed in antisense orientations to oneanother, with some overlap. In order to confirm the expression of both of the transcripts, 5′-and 3′-RACE-PCR analyses were performed for both ORF1 and ORF2 in the WT and HD1. Only a single PCR product was obtained from WT and HD1, and it corresponded to ORF1. This suggests that the LOC_Os01g10460 annotation in the Gramene and Michigan State University Rice Genome Annotation databases may require revision in favor of the Os01t021250 gene from the RAP-DB. Sequencing of the RACE-PCR products revealed 858-bp and a 795-bp cDNA fragments from WT and HD1, respectively, with sequences matching ORF1 (Os01t0201250). Notably, the size of WT cDNA derived from 5′- and 3′-RACE was slightly shorter than that predicted in Os01t0201250 in the RAP-DBby 50-bp and 230-bp at its 5′and 3′ ends, respectively (Fig. 3a).In order to confirm correct transcriptional start site for the gene, we repeated the 5′-RACEanalyses a number of times but consistently obtained only this shorter transcript. According to the transcript structure, the 63-bp deletion in HD1 would then result in truncation of 38 amino acids at the N-terminus of the encoded protein due to translation starting from the second ATG of the open reading frame (Fig. 3a and b). In recognition of the possible derivation of the dominant dwarf phenotype from the mutant allele of the *D-h* gene expressing this deleted transcript, we designated the mutant and the wild-type alleles in this report as *D-h* and *d-h*, respectively.

### Demonstration of the dwarf phenotype conferred by D-h in transgenic plants

**Figure 4:**
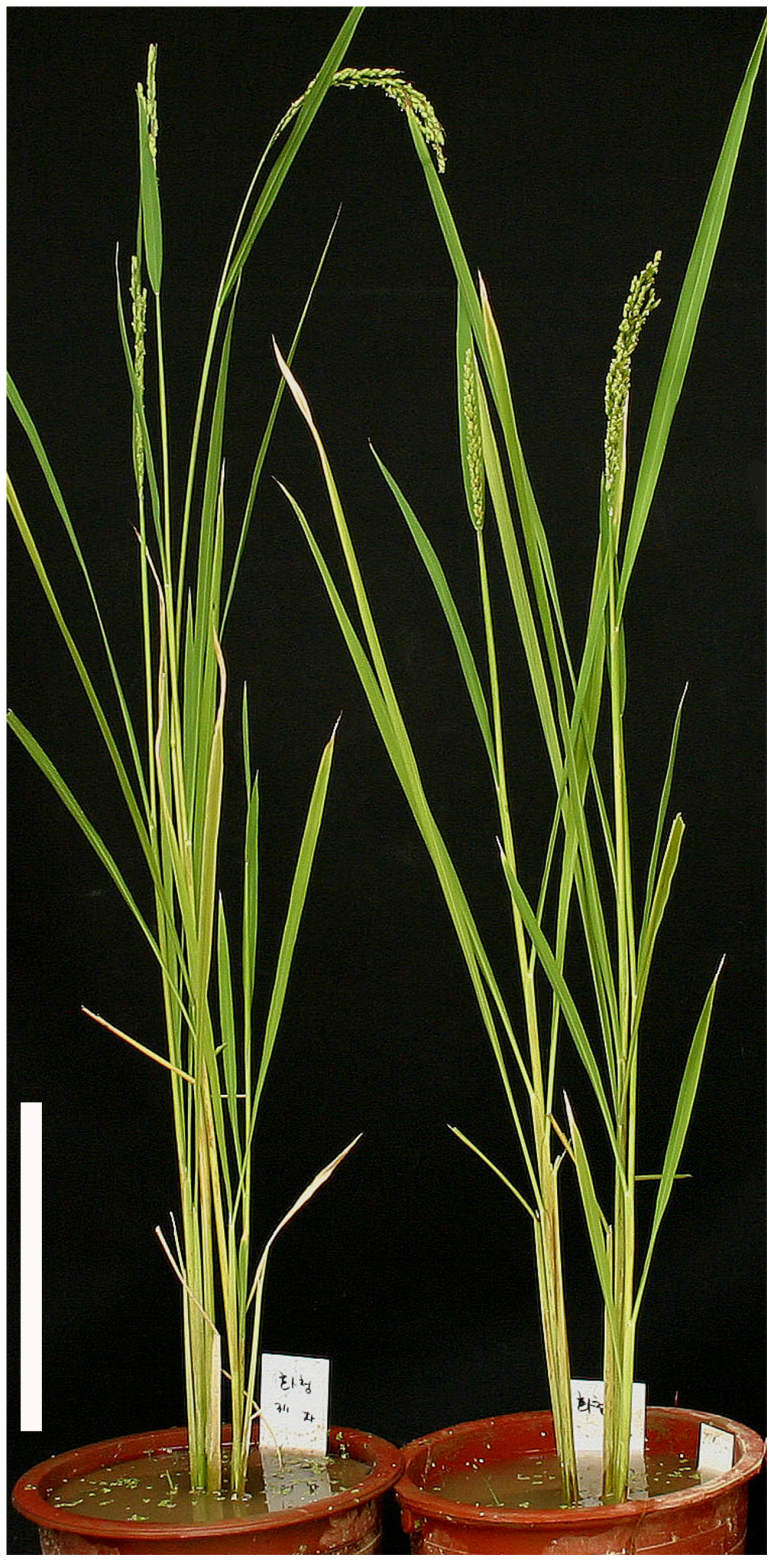
Gross morphology of the overproducing mutant *D-h* protein transgenic plant (right) and WT (left). Bar = 20 cm.

In order to confirm that the 63-bp deletion in the *D-h* genewas indeed responsible for the mutant dwarf phenotype, transgenic rice plants expressing the mutant *D-h* alleleunder the 35S promoter (35S::*D-h*) were generated in the wild-type (WT) Dongjin background (*d-h*) and their phenotypes were observed. All of the transgenic plants showed dwarf phenotypes and smaller grains than those of WT Dongjin (Fig. 4). In contrast, the transgenic Dongjin (*d-h*) plants overexpressing the wild-type *d-h* (35S::*d-h*) or carrying the RNAi-construct of the wild-type *d-h* (RNAi-*d-h*) showed no noticeable change in plant stature or grain size (data not shown). Therefore, it is conceivable that the dominant dwarf phenotype may have been the result of a gain-of-function mutation in the rice *d-h* gene.

### Expression pattern of D-h in the different organs and tissues of rice plants

**Figure 5:**
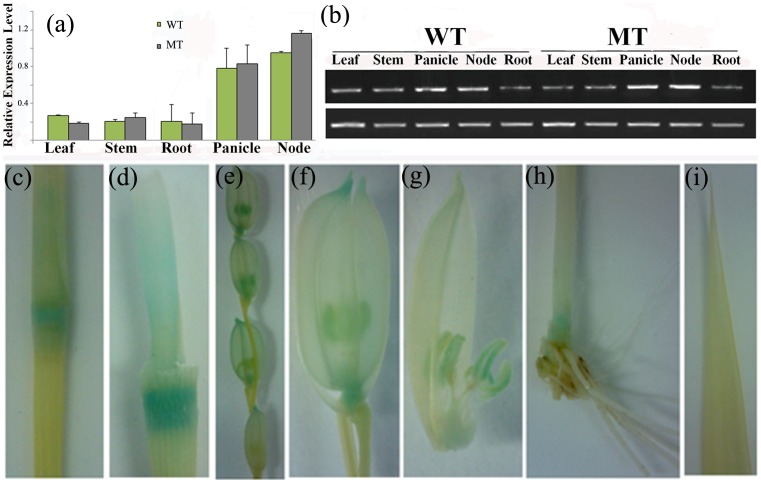
Expression patterns of the *D-h* gene. (a) QuantitativeRT-PCR analysis of the *D-h* gene in organs. (b) Semi-quantitative RT-PCR analysis of the *D-h* gene in organs. (c)–(i) GUS activity detected in the *D-h* promoter::GUS transgenic plants.

In order to better understand the role of the *d-h* gene in rice development, the expression pattern of *d-h* in different organs and tissues was examined using quantitative RT-PCR (qRT-PCR) and a β-*glucuronidase* (GUS) reporter gene under the control of the *d-h* promoter. RNA for qRT-PCR was isolated from mature roots, leaf blades, stems, nodes, and panicles. In both WT and mutant, *d-h* gene expression was most abundant in the panicle and node, with weakly detectable expression observed in the stem, leaf, and root (Fig. 5a and 5b). Corroborating the qRT-PCR results, GUS staining was primarily detected in the panicle, stem base, and node. Relatively strong GUS staining signals were detected in the elongation zone, differentiation zone, and meristematic region of the stem, while no GUS expression was detected in the maturation zone (Fig. 5c and 5d). In the panicle, the GUS signal was found exclusively in the florets, with no detectable expression in the rachis and branches (Fig. 5e–i).

### The d-h gene encodes a novel protein

Based on the 5′-RACE results, the coding sequence (CDS) of the *d-h* gene was predicted to be 354 bp in length with a 235-bp 5′untranslated region (5′-UTR) and a 269-bp 3′untranslated region (3′-UTR), encoding a protein of 117 amino acids (aa). As described earlier, the comparison of the WT *d-h*cDNA sequence with that of the HD1 revealed that the *D-h* allele has a 63-bp genomic deletion. Thiscomprises 31-bp of the coding region and 32 bp of the 5′-UTR, and was predicted to result in a truncated 79-aa protein instead of the 117-aa protein encoded by the WT *d-h* transcript (Fig. 3a and c).

The predicted protein encoded by the *d-h* gene is a novel protein of unknown function with no conserved domains or motifs identified within its amino acid sequences. Orthologs of the rice *d-h* gene were found in several cereal crops, including *Zea mays* (NP_001147534; 90% amino acid identity), *Sorghum bicolor* (XP_002454989; 64% amino acid identity), and *Hordeumvulgare* (BAJ91554, AK360345; 67% amino acid identity) (Fig. 3c).

### Subcellular localization of *d-h* protein

**Figure 6:**
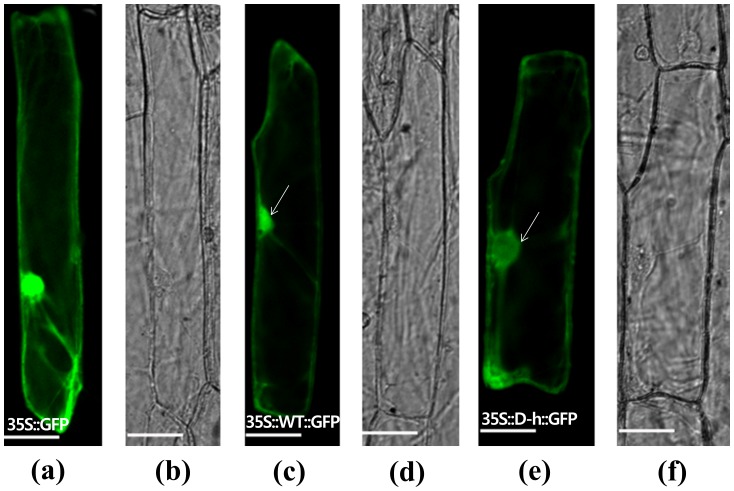
Subcellular localization of the *D-h*-green fluorescent protein (GFP) fusion protein in transformed onion epidermal cells. (a, b) Expression of the control CaMv35S::GFP construct and corresponding DIC image. (c, d) Expression of the CaMv35S::*d-h*-GFP construct and corresponding DIC image. (e, f) Expression of the CaMv35S::*D-h*-GFP construct and corresponding DIC image. Bars = 50 µm.

In order to verify the protein product and to obtain a better understanding of its function, the subcellular localization of the *d-h* protein was examined after generating a chimeric construct of GFPtranslationally fused to the C-terminus of the wild-type *d-h* or the mutant *D-h* gene under the control of the 35S promoter. Upon transfection of onion epidermal cells with these constructs by the particle bombardment method, GFP signals were detected in the nucleus as well as in the cytoplasm for both the wild-type *d-h*::GFP fusion and the mutant *D-h*::GFP constructs, much in the same way as for the GFP control construct. However, the localization pattern of the mutant *D-h*::GFP within the nucleus was slightly differentfrom that of the wild-type *d-h*::GFP fusion protein in that it was a more confined to the inner periphery of the nuclear envelope and had an overall weaker signal (Fig. 6).

## Discussion

### The HD1 is likely to be a GA insensitive mutant

Takeda [26] classified the existing dwarf phenotypes of rice into six groups: N-, dn-, dm-, d6-, nl-, and sh-, in which the dn-type represents those in which all of the internodes are proportionally shortened. The HD1plant described in this study was smallerthan the wild-type plant and showed inhibition of elongation in all of the internodes; therefore, it is considered to belongthedn-type dwarf group. Since the plant hormone GA has been implicated in the regulation of a number of growth and developmental processes, including stem elongation, seed germination, and flowering [4]–[7], a defect in its metabolism and/or signaling is most likely responsible for the dwarf phenotype of the HD1. In our previous publication, the HD1 line was shown to respond similarly to GA_3_ and GA_4/7_as the WT cultivar with respect to the rate of the clum elongation, and was thus unable to fully recover from dwarfism even with GA treatment [27].Those data, taken together with the leaf sheath elongation response to GA and the results of the α-amylase activity test in the present study, suggest that the HD1 is GAinsensitive and fails to convey a proper GA signal. Therefore, it is possible that genes in the GA biosynthetic or signaling pathways might be perturbed in the mutant. To test this, the expression of several selected genes, such as *GA20OX2*, *GA2OX1*, *GA2OX3*, *KO2*, *KS1*, *CPS1*, *GID1*, *GID2*, *SLR1*, and theα-amylase gene *amy1* [6], [28]–[30], was examined using real-time and standard RT-PCR analyses. However, none of the genes, including *amy1*, showed any significant difference in their expression levels between the HD1 and the WT (Fig. S1 and S2); therefore, the precise nature of any GA deficiency or sensitivity in the mutant remains to be determined.. The expression level of copalyldiphosphate synthase (cps), the first enzyme in the GA synthesis pathway, was found to be somewhat lower in the mutant than in the WT (Fig. S1),which might indicatea lower overall amount of GA in the mutant.Conversely, without GA treatment, *GA20ox*appeared to be up-regulated in the mutant, supporting the possibility that the mutant is defective in GA signaling, as transcription of *GA20ox* is controlled by the DELLA protein. However, transcription levels of another Della-controlled gene, *GID1*, did not differ between the mutant and WT (Fig. S2). Therefore, the *d-h* gene might be involved in an unknown GA signaling pathway, which might render the plant carrying its mutant allele insensitive to GA.

### Experimental characterization of the d-h gene locus

Through genetic mapping followed by DNA sequencing, the *D-h* gene was found to be located in a region corresponding to LOC_Os01g10460, a locus for a hypothetical gene of approximately 3 kb in length according to the annotation provided by the latest version (release 6) of the Michigan State University Rice Genome Annotation Database [31]. However the area was annotated differently in the RAP-DB,which predicted an ORF (Os01t0201250) running antisense to LOC_Os01g10460.Ourextensive 5′- and 3′- RACE-PCR analyses revealed that the locus only expresses transcripts corresponding to the ORF Os01t0201250 predicted by the RAP-DB, with no transcription products of LOC_Os01g10460. In order to verify this finding further, we obtained a rice Ds insertion line, YN_16, from the Rural Development Administration (RDA) of Korea, which carries the insertion in the putative proximal promoter region [32] for the expression of LOC_Os01g10460 in the sense orientation. Peculiar phenotypes were not observed in the YN_16 Ds insertion line, and the transcript profile of LOC_Os01g10460 did not differ from that of the WT (data not shown). We also generated transgenic rice expressing the GUS reporter gene under the control of the aforementioned putative promoter of the LOC_Os01g10460 locus, but failed to detect any GUS signals in these plants (data not shown). These results are in contrast to the results of the transgenic plants expressing the GUS gene under a putative promoter for the antisense transcript of LOC_Os01g10460 (Fig. 5). Taken together, our results suggest that the current annotation of the *d-h* gene locus LOC_Os01g10460 in the database is incorrect and should be updated.

Results from 5′- and 3′-RACE-PCR, yielded a shorter transcript than the predicted ORF Os01t0201250 annotated by the RAP-DB. Os01t0201250 predicts a protein of 212 aa, within which a putative VQ motif is found in the N-terminus.The region containing this sequence motif is also highly conserved in several orthologues of the Os01t0201250 in the related crop species including maize, sorghum, and barley (Fig. 3c). VQ domain is characterized with a region of about 57 amino acids of a highly conserved motif and, though speculative, it is thought to regulate plastid gene expression via the modulation of sigma factors, and recently it has been also shown to physically interact with WRKY transcription factors *in vitro* [33]. Interestingly, a VQ motif protein was found to regulate endosperm growth and seed size in *Arabidopsis*, showcasing the domain's functional implication in regulating a number of biological processes [34]. However, according to our 5′-RACE results, the wild-type *d-h* transcript is shorter by 50 bp at the 5′-end than the Os01t0201250's annotation and is predicted to produce a protein of only 117aa. Although our 5′-RACE experiments were repeated several time, is remains possible that we failed to identify the true 5′ end of the *d-h* transcript, especially given that the missing sequences in our version of the transcript are found to be conserved in other orthologs. Thus, although the possibility of two alternative transcripts starting from different initiation sitescannot be ruled out, it is more than likely that our version of the *d-h* transcript does not represent a full-length sequence, althoughthe expressed sequence for full-length Os01t0201250 remains to be identified yetexperimentally.This scenario then immediately raise concerns about validity of other experimental results of ours, particularly the one with transgenic plants expressing the alleged wild-type *d-h*cDNA, but in effect it could have been a mutant version bearing an N-terminal deletion. However,transgenic plants over-expressing the short-form of *d-h*cDNA, as we assumed it was the full-length wild type gene, did not show any peculiar phenotypes, chance of incorrect characterization or misinterpretation of the mutant due to this potential misassumption of the full-length *d-h* gene is rather low. It is intriguing that the transgenic plants expressing RNAi construct of the *d-h* gene did not show any phenotype either, leading us conclude the nature of the *D-h* mutant as a gain-of-function mutant.

### The d-h gene encodes a novel protein that might be involved in GA signaling

Reflecting its importance in crop breeding, more than 60 different dwarf phenotypes have been reported in rice thus far, but only a few of these have been identified as dominant mutants. The gene for the semi-dominant, gain-of-function mutant *Slr-d*, located on chromosome 3, is a consequence of a mutation in the gene encoding the rice *DELLA* protein *SLR1*, which functions as a repressor of GA signaling through an interaction with the GA receptor *GID1* [7]. The gene for another dominant dwarf mutant, *Twisted dwarf 1*(*Td1*), was located on chromosome 11 and was found to encode the α-tubulin protein; this has been recognized as an additional factor that determines plant height through the regulation of microtubule formation [16]. The loci for other dominant dwarf mutants, including *D53*, *Ssi1*, *Sdd(t)*, *Dx*, and *LB4D*, were identified on chromosomes 11, 1, 6, 8, and 11, respectively [13]–[15], [17], [18]. The dwarf phenotype conferred by the HD1was first reported by Koh and Heu [12] as being controlled by a single dominant allele and is associated with shortcompact panicles and smallround grains. In this study, we isolated the *D-h* gene by map-based cloning, which located the gene in a region within the BAC clone AP003197 of chromosome 1. Nucleotide sequencing and RACE-PCR revealed that the WT *d-h* gene is actively transcribed and encodes a protein that is 117 aa in size, has no function that is currently known, and has no conserved domains or motifs (Fig. 3a). Database searches identified the orthologs of *d-h* in other cereal crop species, including maize, barley, and sorghum, and these showed greater than 60% aa sequence identity with one another (Fig. 3c). Functional clues about these orthologous proteins are not available at present, indicating that the *d-h* protein may represent an evolutionarily conserved family of novel proteins that may be involved in a yet-to-be elucidated GA signaling pathway. In the HD1 plant, the *d-h* gene was found to have a 63-bp deletion in the region encompassing part of the 5′-UTR and the first exon, resulting in the production of a truncated protein missing the N-terminal 38 amino acids. By generating transgenic plants expressing this deletion, we confirmed that the mutant gene was directly responsible for the observed dominant dwarf phenotype of the HD1 (Fig. 4). Therefore, it is intriguing to contemplate a possible mechanism by which the mutant *D-h* protein confers a dominant dwarfism phenotype, given that the transgenic rice that either overexpressed or silenced the wild-type *d-h* did not exhibit any abnormal phenotype (data not shown). It is perhaps possible that the deletion in the mutant *D-h* protein may have resulted in the deregulation of a novel signaling pathway operating in response to GA.

The *D-h* mutation was also found to have pleiotropic effects,including short panicles and smallround grains, which may prevent its incorporation into hybrid breeding programs. Nevertheless, the present study provided evidence that the *d-h* gene product plays an important role in regulating cell growth and organ development, possibly through the modulation of novel GA signaling, and may thus be a valuable potential target for agronomic trait improvement. More extensive studies to elucidate the function and molecular nature of the *d-h*mutation gene are planned, such as the identification of possible protein interaction partners for the *d-h* protein.Efforts to better understand the mechanisms by which dwarf-related phenotypes are caused by the *D-h* geneare in progress.

## Supporting Information

Figure S1 **Expression analysis of GA biosynthetic genes by real-time qPCR.** Total RNA was isolated from the WT and the HD1 (MT) plants treated with 10^−4^ GA_3_ solution (WT-G and MT-G) or control solution.
(TIF)

Figure S2 **RT-PCR of GA-inducible genes in aleurone cells.** Fifty embryoless half-seeds were incubated at 30°C for 0, 6, 12, 24, 36 and 72 h in culture medium containing 10^−6^M GA_3_. WT = wild-type, MT = mutant.
(TIF)

Table S1 **PCR-based molecular markers used for fine mapping of the** ***d-h*** **gene.**
(DOCX)
