# Supplementary figures and images for: Isolation and Characterization of a Dominant Dwarf Gene, D-h, in Rice
Source: PLoS One. 2014 Feb 3;9(2):e86210. doi: 10.1371/journal.pone.0086210 (PMC3911911; doi:10.1371/journal.pone.0086210)

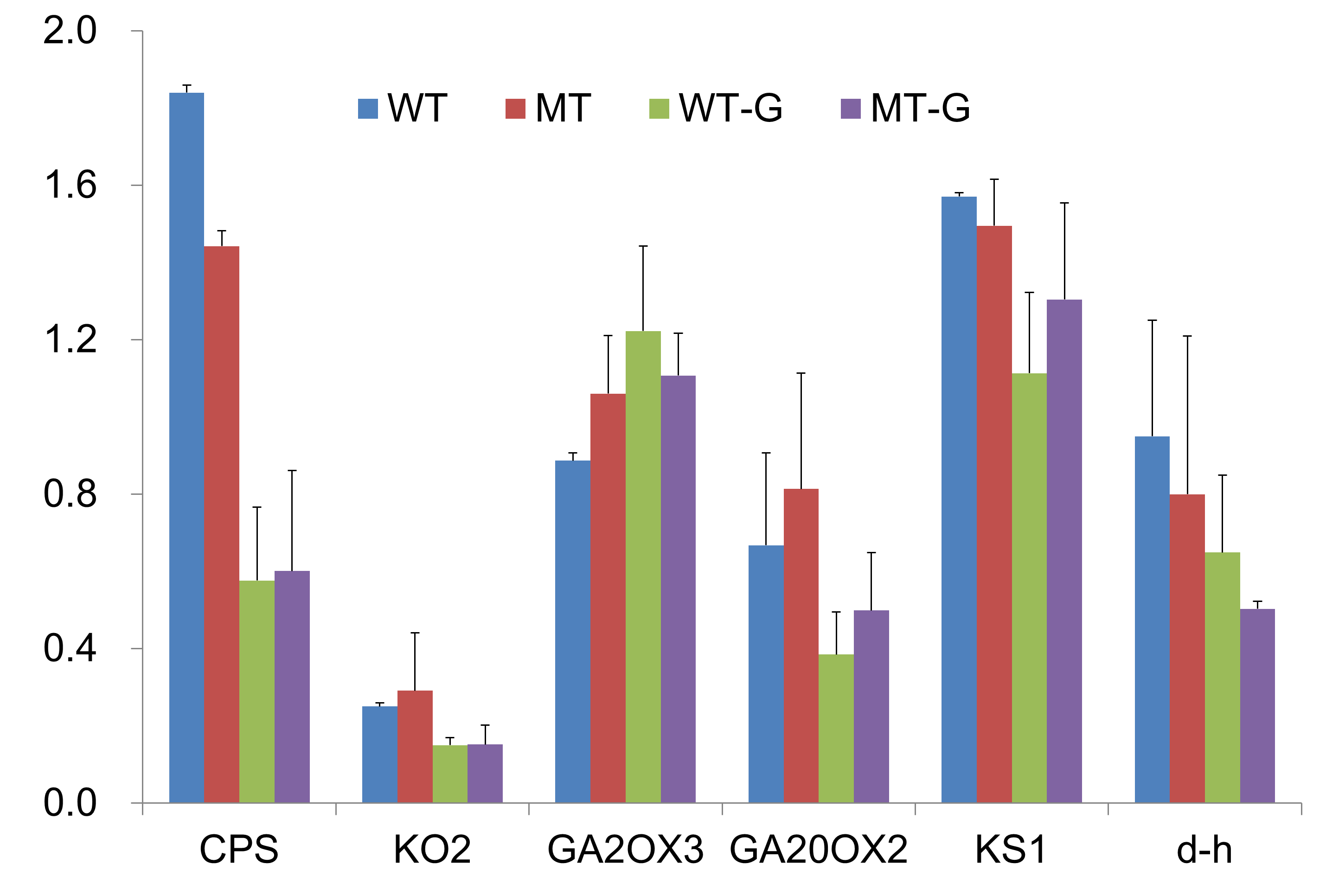

Supplement: Figure S1 — Expression analysis of GA biosynthetic genes by real-time qPCR. Total RNA was isolated from the WT and the HD1 (MT) plants treated with 10−4 GA3 solution (WT-G and MT-G) or control solution. (TIF) [file pone.0086210.s001.tif]

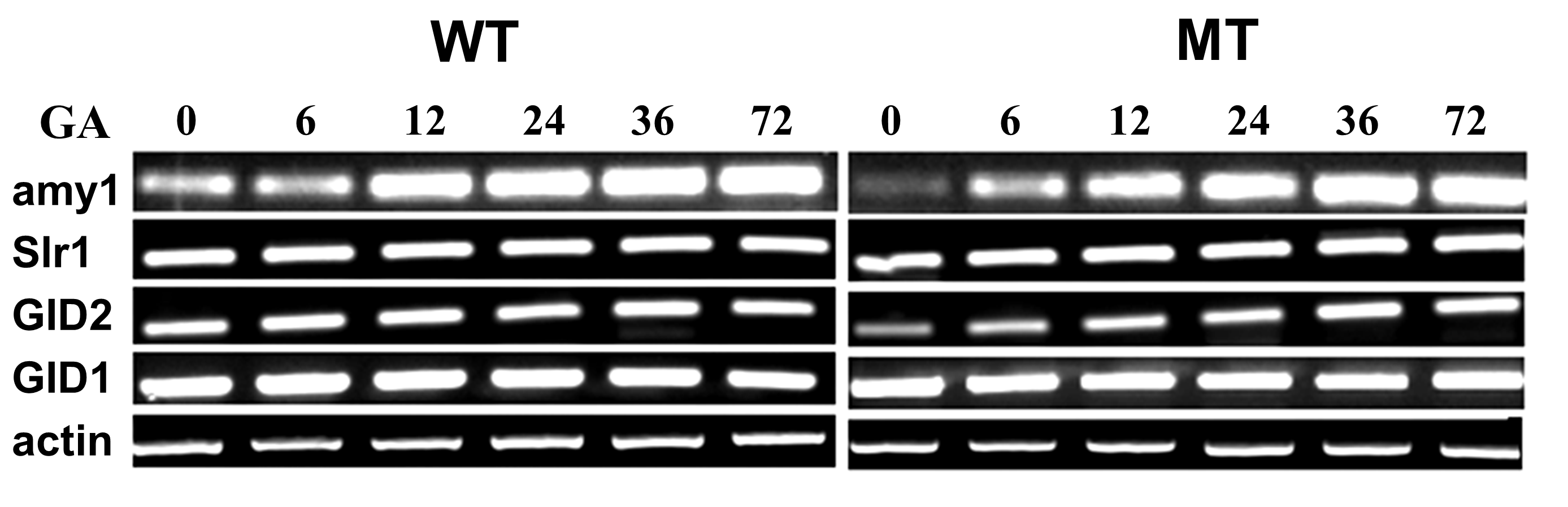

Supplement: Figure S2 — RT-PCR of GA-inducible genes in aleurone cells. Fifty embryoless half-seeds were incubated at 30°C for 0, 6, 12, 24, 36 and 72 h in culture medium containing 10−6M GA3. WT = wild-type, MT = mutant. (TIF) [file pone.0086210.s002.tif]
