# Supplementary material for: Isolation and Characterization of a Dominant Dwarf Gene, D-h, in Rice
Source: PLoS One. 2014 Feb 3;9(2):e86210. doi: 10.1371/journal.pone.0086210 (PMC3911911; doi:10.1371/journal.pone.0086210)
Supplement: Table S1 — PCR-based molecular markers used for fine mapping of the d-h gene. (DOCX) [file pone.0086210.s003.docx]

**Table S1**. PCR-based molecular makers used in fine mapping of the d-h gene

| STS marker | Forward primer (5’- 3’) | Reverse primer (5’- 3’) |
| --- | --- | --- |
| 1013.1-1 | tttctttagctccgccttga | ctttcgcacaaggacgtg |
| 1022.6-1 | catggatgatgcttccctct | ttgacagtggctccacaaag |
| 1024.0-1 | TTCAGAGCAGCAGAGCATGT | TTTTTCACCATTTCTTTGCCTTA |
| 1027.3-2 | GTGCTGTTCCATGCTCCAC | ggggaggggtTAAGATGACT |
| 1027.3-1 | agcggtagtaccatcggaga | agtacagatggaaaatccaacg |
| AP3105-1 | ttttaggacggaggaagtaactttt | TGGTGGAGAATTGTGATTGA |
| D-h-4 | TTGGCAAGAAAAGTTGACCA | CCAAACATTCGTTTCTGAGC |
| D-h-3 | AAGCTTATCCTGCCACTTTTT | TTTTGCATGTCTCCCTTTCC |
| AP3197-1 | agccgagttagcatttcagc | TTCTTCACGAGCACACATCC |
| 1028.4-3 | TCCTCGAGATTGGAGACAGC | CTCCAAATGCCGAGAAAATC |
| 1028.9-1 | GATGAAACTCTGGGCAGGAA | CAGGAAACCCAAAATCCTGA |
| 1038.8 | aagtcttgcagacgacacca | caaagctgccactgatgaaa |
| InDel | GGAAGGGTGGAGGTAGAACC | TCTCTCTCTCCCTTGCCAAA |
